# Editorial: Impact of COVID-19 on the detection and control of chronic non-communicable diseases: repercussions on the sustainable development agenda

**DOI:** 10.3389/fpubh.2024.1397176

**Published:** 2024-04-03

**Authors:** Alvaro Francisco Lopes Sousa, Thereza Maria Magalhães Moreira, George Jó Bezerra Sousa

**Affiliations:** ^1^Hospital Sirio Libanes, São Paulo, Brazil; ^2^Department of Nursing, State University of Ceará, Fortaleza, Ceará, Brazil; ^3^Ministry of Health, Brasília, Brazil

**Keywords:** COVID-19, Sustainable Development Goals, surveillance, 2030 Agenda, non-communicable chronic diseases

According to the World Health Organization (WHO), the COVID-19 pandemic surpassed the health boundaries and had devastating social, educational, and economic consequences. Globally, it has caused 774.49 million cases and 7.03 million deaths until February 4th, 2024 (1). The repercussions in detecting and controlling Non-Communicable Diseases (NCDs) are one of the most worrying issues regarding the COVID-19 pandemic, which may have considerable implications for the 2030 Sustainable Development Agenda (2).

The COVID-19 pandemic increased pre-existing disparities as well as worsened the global capacity to control and prevent NCDs. Generally, access to essential health services for people with diabetes, hypertension, obesity, cardiovascular diseases, and cancer, among other diseases was reduced by social isolation, fear of infection, and lack of availability due to an overload of health systems (3). In order to understand the access to blood donation services during the pandemic, Kim and Cho highlighted the environment as one important predictor.

Analysis of the intricate interplay between COVID-19 and NCDs involves recognizing the complex vicious cycle that worsened global public health in the last years and exacerbated risk behaviors that directly contributed to the prevalence of NCDs. In general, several factors can be attributed to this phenomenon, from psychological stress to prolonged social isolation, and lifestyle changes resulting from containment measures imposed to control the viral circulation. By aiming to measure preventative behaviors related to COVID-19, Bajamal and Alazani have developed a Knowledge Attitudes and Practice (KAP) instrument for the Arabic population.

Moreover, the consumption of tobacco, alcohol, and other drugs significantly increased during the pandemic and many individuals used these substances to relieve stress and anxiety created by uncertainty, social fatigue, and economic difficulties (4). Likewise, changing dietary habits toward processed, nutritionally deficient foods and reduced physical activity due to the closure of public spaces and fitness facilities enhanced risk factors for NCDs (5).

Besides, NCDs play a critical role in increasing vulnerability to severe forms of COVID-19. Individuals with preexisting conditions have greater chances of presenting severe complications of the disease and hospitalization for intensive care, which ultimately enhances death risk. Part of this occurs due to NCDs altering immunity and decreasing capacity to fight SARS-CoV-2 infection. In this sense, Trecarichi et al. have identified predictors of hospital mortality in four different COVID-19 waves and, among those, many were related to NCDs, which reinforces the relevance of this Research Topic.

In general, both this scenario and the interplay between the diseases put at risk the achievement of the Sustainable Development Goals (SDG). Among them, the ones related to health and wellbeing (SDG 3), reducing inequalities (SDG 10), and climate change (SDG 13) invite us to a decisive decade of action to reach the targets of the 2030 Agenda. The pandemic revealed the weaknesses of health systems in the world and the need for an intersectoral and resilient approach to public health, including social, economic, commercial, and environmental determinants of health (Arscott et al.).

The COVID-19 pandemic triggered a series of events that will affect global health for several years, mostly in people with NCDs. A detailed comprehension of how the pandemic will impact the population's health in the long term is paramount for both civil society and the scientific community, mostly because of the already known burden of NCDs within global health systems. Furthermore, the pandemic revealed and enhanced already existing inequalities, underscoring the critical role of different sectors in public health. These actions are vital to health promotion once they link determinants directly related to health but also indirect factors such as economic and environmental conditions. In this regard, Zhou et al. have identified environmental factors that influenced the number of COVID-19 cases as a proposition for sustainable planning action that surpassed the health sector.

Therefore, the need for sustainable global surveillance systems for NCDs has never been more evident than in the post-COVID-19 era. These systems have to be able to monitor the risk factors for increased incidence and prevalence of NCDs as well as health outcomes. This is particularly relevant in low- and middle-income countries where resources for the prevention and treatment of NCDs are limited, and managing risk factors is hindered due to financial restrictions, lack of infrastructure, and educational challenges.

In order to improve risk factor surveillance in these regions is necessary to significantly invest in research, resources, and education along with a collaborative and integrated approach by involving governments, the private sector, and local communities. Such actions will help to identify and mitigate risks to NCDs and will provide data to guide public health policies and funds allocation.

Aligning these efforts with SDGs is crucial because they offer a wide framework to approach the affairs related to global health and wellbeing, including poverty eradication, guarantee of quality education, and promotion of healthier lifestyles. By integrating surveillance and management of NCDs in the framework, it is possible to improve health outcomes in people living with these conditions and move toward a more equal and sustainable world.

Finally, it is important to note that the development and implementation of these surveillance systems and mitigation strategies require long-term perspective, political commitment, and international cooperation. The COVID-19 pandemic highlighted the importance of connecting global health, sustainable development, and equity. Now, it is essential to adopt a collaborative approach to face the challenges imposed by NCDs in the post-COVID-19 era. Therefore, in a post-COVID-19 era, we highlight that the conjoint effort union from both public and private sectors with civil society, and the scientific community may reach a world more sustainable, equal, and with a lower burden of disease by 2030.

## Author contributions

AS: Conceptualization, Writing – original draft, Writing – review & editing. TM: Writing – original draft, Writing – review & editing. GS: Conceptualization, Writing – original draft, Writing – review & editing.
